# Adjust your own oxygen mask before helping those around you: an autoethnography of participatory research

**DOI:** 10.1186/s13012-020-01002-1

**Published:** 2020-09-03

**Authors:** Abby M. Steketee, Thomas G. Archibald, Samantha M. Harden

**Affiliations:** 1 Department of Human Nutrition, Foods, and Exercise, Virginia Tech, 1981 Kraft Drive, Room 1032, Blacksburg, VA 24060 USA; 2 Department of Agricultural, Leadership, and Community Education, Virginia Tech, 284 Litton-Reaves Hall, Mail Code 0343, Blacksburg, VA 24061 USA

**Keywords:** Implementation, Novel methods, Epistemology, Knowledge translation, Context, Engagement, Qualitative research, Anthropology

## Abstract

**Background:**

There is a need to unpack the empirical, practical, and personal challenges within participatory approaches advocated to optimize implementation. The unpredictable, chaotic nature of participatory approaches complicates application of implementation theories, methods, and strategies which do not address researchers’ situatedness within participatory processes. As an implementation scientist, addressing one’s own situatedness through critical reflection is important to unearth how conscious and unconscious approaches, including ontological and epistemological underpinnings, influence the participatory context, process, and outcomes. Therefore, the aim of this exploratory work is to investigate the heretofore blind spot toward the lived experience of implementation researchers within the participatory process.

**Methods:**

We developed an integrated research-practice partnership (IRPP) to inform the implementation of a gestational weight gain (GWG) control program. Within this IRPP, one investigator conducted a 12-month autoethnography. Data collection and triangulation included field notes, cultural artifacts, and systematic timeline tracking. Data analysis included ethnographic-theoretical dialogue and restorying to synthesize key events and epiphanies into a narrative.

**Results:**

Analysis revealed the unpredicted evolution of the GWG program into a maternal health fair and three themes within the researchers’ lived experience: (1) permeable work boundaries, (2) individual and collective blind spots toward the ontological and epistemological underpinnings of implementation paradigms, and (3) maladaptive behaviors seemingly reinforced by the research culture. These themes contributed to the chaos of implementation and to researchers’ experience of inadequate recovery from cognitive, emotional, and practical demands. These themes also demonstrated the importance of contextual factors, subjectivity, and value-based judgments within implementation research.

**Conclusion:**

Building on extant qualitative research guidelines, we suggest that researchers anchor their approach to implementation in reflexivity, intentionally and iteratively reflecting on their own situatedness. Through this autoethnography, we have elucidated several strategies based on critical reflection including examining philosophical underpinnings of research, adopting restorative practices that align with one’s values, and embracing personal presence as a foundation of scientific productivity. Within the predominant (post-) positivism paradigms, autoethnography may be criticized as unscientifically subjective or self-indulgent. However, this work demonstrates that autoethnography is a vehicle for third-person observation and first-person critical reflection that is transformative in understanding and optimizing implementation contexts, processes, and outcomes.

Contributions to the literature

- This work suggests that theory-based autoethnography (i.e., personal narrative) is a thought-provoking, action-inspiring method for capturing implementation processes and outcomes, particularly within participatory research.
- In recognizing how their own values, beliefs, and behaviors influenced the project at hand, researchers identified several strategies for thriving in the chaos of implementation.
- These strategies emphasize reflection, well-being, and presence as tools toward personal, professional, and scientific clarity.

## Background

In translating research findings to real-world benefit, participatory research [1–4] purportedly embodies the spirit of a quote often attributed to cultural anthropologist Margaret Mead: “Never doubt that a small group of thoughtful, committed citizens can change the world: indeed, it is the only thing that ever has” [5] (p. 111). As a vehicle for change, participatory research is characterized by active collaboration between researchers and diverse stakeholders [1]. This collaboration is intended to empower stakeholders—specifically those who are underrepresented in research decision-making—to shape all aspects of research, ultimately optimizing how a program or policy works within stakeholders’ unique lives and communities [4, 6]. Overall, participatory research holds promise as the backbone of pragmatic [7] implementation [8] to promote feasible, user-friendly applications of research findings to the daily lives of community members.

However, as participatory research has become more common, experts have uncovered a “dark side” to this collaborative model with practical, personal, and professional costs to researchers, as well as costs to stakeholders, the research itself, and research as a profession [9]. Ample evidence suggests that participatory research poses people-oriented, emotionally charged challenges plus complicated logistics and scheduling barriers in constant flux [10–14]. Exemplifying the emotional dimension of these challenges, researchers in recent focus groups described pursuing dissemination and implementation (D&I) science as “intimidating” and “career suicide” [15]. Furthermore, within participatory processes, researchers may have to navigate conflict and tension related to organizational change and feedback loops, particularly when there are power differentials between stakeholders (e.g., physician and patient) [10]. The unpredictable nuances of working within complex human environments can be chaotic [10], challenging the usability of current implementation theories, methods, and strategies [16, 17] that do not specifically acknowledge researchers’ roles or intrapersonal resources to initiate and maintain participatory processes [18].

In addition, the uncontrollable, value-laden nature of a participatory approach [19, 20] seems incommensurable [21, 22] with positivist or post-positivist paradigms reflected in mechanistic implementation advancements such as quantification of discrete implementation activities [23] and calls for deductive qualitative research [24]. In general, (post-) positivist paradigms emphasize systematic a priori hypothesis testing, experimental control, elimination of bias, generalizable predictions, and research quality determined by conventional internal and external validity aimed at explanation. In contrast, constructivist (sometimes called interpretive) paradigms emphasize the subjective nature of knowledge, naturalistic inquiry with participant voice, reflexivity, iterative theorizing, and research quality determined by trustworthiness aimed at understanding meaning [19, 25, 26]. For additional description of scientific paradigms, see Table 1 in Greenhalgh et al. [26] or Table 6.2 in Guba and Lincoln [19].

**Table 1:** Results of the ethnographic-theoretical dialogue

**Theme #1: Permeable Work Boundaries:** “As I left [coffee shop], I realized that I’d just added another person into my internal carousel of thinking and feeling. I put myself in a position to be more responsive to more people. It’s increasingly difficult to protect uninterrupted time to do my ‘intellectual’ academic and research work from the boundless work of caring about something. And then it gets harder and harder to stop the intellectual work of figuring something out from flowing into dinner with Steve, walking with Bo, going to sleep. There’s not an on/off switch for these intangible dimensions of cognition and emotion” (May 15, 2019).		
*SUBTHEME DESCRIPTION*	*ETHNOGRAPHIC EVIDENCE*	*THEORETICAL/LITERATURE DIALOGUE*
**Constant digital connectivity:** The GRA’s use of email blurred the lines between work and home. Opportunity for Progress: Intentional email and social media use	• “It’s the third time this week that I’ve let an email that arrives late in the afternoon become a **cascade of things to work on** and send back and forth through dinner and reading for class. So, now I’m amped up, like I just finished an interval workout on the treadmill. Steve is already asleep, but I’m wired: First, I might get another follow-up email that “needs” to be answered. Second, I’m adrenalized from what feels like “firefighting.” Third, I’m unsettled since I didn’t do the neuro reading. Fourth, I’m mad at myself for getting caught up in the frenzy, blowing things out of proportion, and not regulating my own perspective and reaction. All because of my conscientiousness—or should I say guilt, obsessiveness, **drive—toward answering email 24-7**” (February 19, 2019).	• Individuals in academic and creative roles may experience emails as disruptive, exacerbating **stressful perceptions of lack-of-control** [27]. • Email can contribute to **stress and overload** [28, 29], the sensation of doing more things without actually getting anything done. • Email may prompt **multi-tasking** which is antithetical to performance and productivity [30, 31], as well as to deliberate, uninterrupted concentration needed to master challenging tasks [32–34].
**Determination and burnout:** Despite years of coaching elite athletes on the importance of recovery, the GRA realized that she was sacrificing her own well-being and work capacity as the research-academic culture that flaunts busy-ness fanned her inner fire to succeed in producing high-quality work deemed significant by faculty, the implementation field, and the real-world. Opportunity for Progress: Strategic recovery to prevent burnout and emotional exhaustion.	• “If I post this video of me practicing sun salutations, are people going to think that I’m not working hard? Even though I know it’s ridiculous to **feel guilty** over not posting something about ‘only 2.5 hours of sleep, Starbucks, and Altoids,’ here I am—feeling like an impostor in this research/grad school life” (October 20, 2018). • “Just look at Tom Brady and Under Armor pajamas: **In elite athletics, recovery is a big deal**. When I was coach, getting the most determined swimmers to take time away from the pool was one of my biggest challenge. But here in academia, no one is coaching graduate students or co-workers to stay sharp by incorporating recovery. No one is even encouraging them to stay home when they’re sick. My advisor told me that one of her colleagues—or maybe it was one of her professors—said that she didn’t care what the personal excuse was, **the work still had to get it done**” (November 16, 2018). • Attending the 11^th^ Annual D&I Conference: “I didn’t need a randomized controlled trial based on an intricate framework to conclude that quite a few health researchers aren’t that healthy. Talking about the next glass of red wine, glancing between a laptop and cell phone propped on their knees during a keynote lecture, feeling guilty about missing a lecture to squeeze in a spinning class. Are **our minds so full** to do innovative, respected work that we can’t be **mindful of our own health**?” (December 8, 2018).	• There’s no literature on burnout among implementation researchers, but in two studies published in 2019, more than one-third of scientists in health-related fields reported **burnout** [35, 36]. • Long hours characterize academia and research [37, 38], even though meta-analysis indicates that working more than 55 hours per work is associated with an **increase in the risk of incident coronary heart disease and incident stroke** compared to working 35-40 hours per week [39]. • The **Effort-Recovery Model (ERM)** from industrial and organizational psychology suggests that inadequate recovery from work demands may deplete an individuals’ psychological and physiological resources to accomplish what has to be done in a certain place within a specific time frame [40]. Recovery is an essential process to restore one’s capacity to meet work demands [41–43]. See below for additional dialogue related to recovery.
**Emotional work**: The GRA’s personal connection to IRPP members, prenatal yoga attendees, and maternal health expo volunteers included an emotional dimension with authentic trust and empathy that led to both laughter and heartache within the lived experience. Opportunity for Progress: Expanded D&I competencies that include social-cognitive dimensions	• After learning about the **loss** of community member’s partner: “I usually smile at the viridescence of the moss finding its way through the gray cement blocks. But today it’s garish. The moss is living, but KK’s wife is not” (June 6, 2019). • After the parents of an IRPP member **suffered** a series of health events: “I woke up thinking about how SM will feel moving her parents into the retirement home…that shift from your parents taking care of you to you taking care of your parents…reminds me of the first time I realized that when Pops and I walked into the ocean holding hands, I was stabilizing him, preventing him from getting knocked over by the waves, not him holding up me anymore” (July 11, 2019).	• Qualitative researchers in various fields acknowledge the impact of **compassion stress** [44]**, empathy** [45]**, and emotional labor** [46, 47] as participant-researcher relationships develop. • To the investigators’ knowledge, accounts of participatory research in D&I science have not explored this emotional component of researchers’ **positionality** as an insider in the implementation process. • Demonstrating our current **blind spot toward the emotional dimension** of our work, fellows who enrolled in the Mentored Training for Dissemination and Implementation Research in Cancer (MT-DIRC) program ranked emotional support as their lowest priority for mentoring [48]. In addition, published educational competencies for D&I science do not include any social-cognitive dimensions, even under practice-based considerations [49]. • Emotional and psychological processes erode recovery. Replaying a stressful event in one’s mind—“**perseverative cognition”**—affects the cardiovascular, immune, endocrine, and neurovisceral systems even if a person is physically separated from the stressor [50, 51]. • For example, **work-related rumination** (i.e. thinking about an element of one’s job during off-hours) is associated with reduced cognitive functioning [52] and co-worker related helping behaviors [53].
**Theme #2: Blind spots or conflicts within scientific paradigm:** “It seems that D&I scientists can experiment with a different wavelength of thinking that transcends the replicable methods, visible research process, and generalizable frameworks. Something deeper, something that takes courage, something like that adage about ‘a ship is safe at shore, but that’s not what ships are built for’” (December 27, 2018).		
*SUBTHEME DESCRIPTION*	*ETHNOGRAPHIC EVIDENCE*	*THEORETICAL/LITERATURE DIALOGUE*
**Feasibility of pragmatic methods with positivist/postpostivist underpinnings:** The GRA’s academic classes did not delve into philosophical underpinnings of scientific methods. Sensing that something was missing, she independently read about the ontological and epistemological foundations of various scientific paradigms. In her interpretation, the positivist/postpositivist evidence preferred by the implementation field is at odds with calls for actionable, stakeholder-centered approaches. Opportunity for Progress: Reflexivity of how philosophical underpinnings may curtail novel approaches	• After a seminar: “Putting meta-analyses and systematic reviews at the top of the **evidence hierarchy applies to a conventional scientific paradigm** of generalizable quantification across the translational spectrum. Perhaps a meta-analysis is the best thing for clarifying a cause-effect relationship between bone density and vitamin D supplementation, something that has a literal dose. But using meta-analysis to pinpoint the effects of a community advisory board would be like showing someone the electromagnetic spectrum to describe the taste of the cold orange I ate at hour 28 of the dance marathon” (October 13, 2018) • Applying lessons from the D&I class to writing a methods section for a community advisory board: “If the main criteria for judging the significance of a finding is its internal and external validity, then what’s the point of community work that inspires change or empowers people or embraces subjective experience? The potentially expansive impact of pragmatic **community work challenges narrow definitions**—or perhaps purposes—of science” (November 14, 2018).	• Calls for pragmatic approaches entail research **methods and outcomes that matter** to stakeholders in real-world settings [7, 54]. Yet, qualitative research within implementation science is “positivist and deductive in nature, with their use increasingly guided by theories and organized by one or more implementation models or frameworks” p. 4 [24]. There seems to be a **tug-of-war** between finding out what stakeholders value through genuine participatory research versus employing a methodology that is rapid and geared to specific issues determined *a priori*. • In other words, it seems that we are trying to employ seemingly **value-free methods to achieve real-world value.** • Scientific paradigms are human constructions [19, 54]. Paradoxically, our deeply-held values as scientists influence our drive to be objective and value-free. • Examination of the ontological (*what is the nature of reality?*) and epistemological (*what is the nature of knowledge*) **foundations of the evidence that we’re willing to accept as credible** [19, 25, 55, 56] may facilitate our ability to be truly pragmatic and stakeholder-centered, acknowledging the role of values in capturing what really matters to stakeholders. • Overall, it may not be **feasible** to simultaneously achieve scientific rigor characterized by systematic objectivity and real-world relevance characterized by practical meaning [8, 57]. Participatory research that strives to accomplish both may actually accomplish neither, having tepid significance scientifically, practically, and personally.
**Incongruence of a prior/systematic design within participatory process:** The GRA grappled with the need for detailed *a priori* methods sections for grant applications and dissertation proposals, even when investigators intend the direction of the research—including outcomes—to truly develop from IRPP input within a participatory approach. Opportunity for Progress: Funding avenues for the invisible relational work in participatory research	• Trying describe her method for finding community members to teach seminars at the Mom Expo: “I’m trying to write down how I will *systematically* go about ‘recruiting’ educators for the Mom Expo so that the process could be *replicated*. The general action of genuinely listening to a person in an environment in which she is comfortable can be replicated on some level, but there’s not an objective, **systematic equation for building true trust.** Just like recruiting collegiate swimmers, connecting with community members takes time and emotional energy but it can’t be distilled into something objective and traditionally ‘scientific.’ The distilled part, the **replicable part is love** and “humbition” and I don’t think that’s going to fly in the methods section” (February 13, 2019).	• Successful **D&I grant writing** requires well-reasoned clarity with pre-established collaborators and benchmarks [58]. In addition, grant writing for the National Institutes of Health (NIH) requires “strict application of the scientific method to ensure robust and unbiased experimental design, methodology, analysis, interpretation, and reporting” (NIH Grant Writing handbook, page 16). • These descriptions of what it takes to win a grant suggest that participatory research is not fundable—at least not participatory research that leaves room for naturalistic networking, authentic exploration, and iterative enactment of what stakeholders—like pregnant women—really value. • Currently, the networking and relationship-building inherent in participatory research appears to be **“invisible work”**—time consuming but unrecognized [59–61]
**Lack of researcher reflexivity/situatedness/positionality**: In leading the IRPP, the GRA found that what she had learned in various leadership trainings was not common in implementation science. There appeared to be no training, guidelines, or frameworks about how to leverage her own leadership style and personality to optimize the participatory approach. Other implementation researchers she met through the year were also directly leading participatory efforts involving stakeholders but had received no leadership training or instruction about the importance of intrapersonal capacity. Opportunity for Progress: Leveraging researchers’ personality strengths and honing leadership skills	• “There were mentions of **soft skills** at D&I [11^th^ Annual Conference], but the D&I competencies from the [D&I graduate school] class syllabus don’t reflect how those soft skills might really matter” (December 8, 2018). • “The first thing we did in the Energizing People for Performance Executive Education program [at Northwestern University’s Kellogg School of Business] was take various assessments to **understand our own behaviors, communication, and personality as a centerpiece to effective leadership**. My teams swam much better after I realized that my intense focus was unintentionally intimidating to my athletes and that my analytical, perfectionistic tendencies slow down the process of doing anything” (December 27, 2018). • “Suddenly [in running labs, collaborating in team science, leading meetings with stakeholders], researchers who have grown up (in school, etc.) being praised for accuracy, analytical skills, depth of knowledge, and ability to navigate peer-reviewed literature have to build spontaneity and easy-going attitudes that work for relationship-building and navigating real-time personal situations. It’s like when I needed to switch from the athlete mindset of staring at the line in the bottom the pool to the coaching mindset of connecting with people. I didn’t have to fundamentally change my core self, I just had to **develop awareness** of my ingrained focused and working style” (February 22, 2019). • After listening to a podcast of Graham Duncan describing how our greatest **strengths are adjacent to our greatest dysfunctions**: “So now I’m feeling like an Aristotelian tragic hero: Just call me Oedipus Rex, Phd wannabe implementation extraordinaire. The determination that that earns me praise and the very GRA position that I currently hold is the same determination that causes this eye twitch from standing in front of the computer long after I’ve lost the edge of creativity and insight” (March 16, 2019).	• **Personality traits** are related to leadership styles [62, 63] and leadership styles influence [64] team performance including communication and cohesion [62, 65–67]. • The influence of researchers’ **leadership styles** and personalities on team science [68] and participatory research have not been studied, even though both approaches are advocated. We know little about implementation researchers’ leadership styles and personalities, including their effects on the participatory process. • However, research indicates that scientists—like all humans—have **adaptive and maladaptive personality facets** such as self-critical perfectionism related to perseverative cognition and inadequate recovery [69]. • On the adaptive side, researchers may also have high levels of **openness** which is the capacity for change, variety, and novelty [70]. Openness is positively associated with creativity, number of citations, and *h*-index but not number of publications [71]. (See discussion of publish or perish below.) • Understanding their own personalities and strengths could help researchers prevent fatigue and burnout by offsetting the **ego depletion** that occurs when we reach the end of our willpower [72, 73]. In other words, constantly keeping natural personality traits in check or extending into areas that don’t suit our personal values could deplete intrapersonal resources related to cognitive performance (e.g. attention) and general quality of life.
**Theme #3: Research/Academia Culture:** “I’m constantly reinforcing the very culture that stresses me out and slows the translational process by working toward peer-reviewed publication. In other words, the assignments I receive and the assignments I strive to do as a D&I grad student undermine dissemination; pouring my time into crafting information for journals and grad committees is necessary to earn my degree and paycheck, but it’s an energy suck away from actually getting information to the public” (February 23, 2019).		
*SUBTHEME DESCRIPTION*	*ETHNOGRAPHIC EVIDENCE*	*THEORETICAL/LITERATURE DIALOGUE*
**Pressure to “publish or perish”:** The GRA felt torn between reinforcing the current paradigms of productivity versus pursuing transformational dissemination by bucking the current focus on journals and repositories. Opportunity for Progress: Optimize motivation and achievement orientation to improve research quality and impact, instead of overemphasizing publication quantity	• “One reason I left elite athletics was to get away from the **obsession with outcomes**. But with the language here—**bean counting**, line on the CV—I feel submerged in a culture that quantifies personal worth in productivity stats like number of publications. If I hadn’t spent years disentangling my self-worth from the time on a stopwatch, I would be probably be unconsciously engrossed in this system of constantly proving I’m *enough* by publishing something” (April 30, 2019). • “It’s clear through all of the discussions at [prenatal] yoga that women don’t want to be a research subject; they don’t want the mom expo to feel like an experiment. So I’ve got this **choice**—**prioritize their values OR prioritize peer-reviewed journal values.** They’re not mutually exclusive, but designing a study for people who don’t want to be in a study is different from designing a study for people who publish journals and approve dissertations…I need to distinguish between a service mindset and manuscript obsession” (February 26, 2019).	• The ubiquitous pressure to publish or perish has been called a perverse incentive, influencing funding, professional advancement, and even scientific trustworthiness [74] • On the spectrum from extrinsic to intrinsic motivation, pressure to publish may foster “controlled” types of **motivation** associated with burnout, poorer performance, and overall well-being [75–79]. • Pressure to publish also reflects a “performance” **achievement orientation** that is based on proving one’s competence in comparison to others, as opposed to a “learning” or “mastery” achievement orientation that is based on personal growth regardless of comparison to others [80–82].
**Norms of multiple roles and responsibilities:** The GRA observed her professors juggling teaching, research, and service responsibilities and learned about PhD career trajectories. She also read many online bios of implementation researchers and noticed the extensive email signatures. Even though her dissertation committee encouraged her to commit to one deep line of research inquiry for the dissertation, she felt that academia, implementation science, and participatory research demand individuals to spread themselves across multiple domains and responsibilities. Opportunity for Progress: Streamline responsibilities and time commitments to reducing competing demands	• After reading guidelines for **earning tenure:** “It’s as if tenure-track faculty are expected to be the coach, be the swimmer, and be the referee all at one time” (February 20, 2019). • Reflecting on email signatures: “So. **Many. Positions.** (And acronyms). It’s the opposite of athletics. In athletics, individuals get more and more specialized in one sport or one position in a sport as they become more elite: They channel their energy and invest their training time into specific sports which is why it’s so rare to have multi-sport Olympians or even Division I athletes. But in academia, it seems that the road toward ‘eliteness’ requires individuals to **take on responsibilities in broader and broader realms**—to **survive** doing as many things as possible as opposed to doing one or two things really, really well” (May 15, 2019). • “It’s hard to take the reins of a new project when your hands are already full” (scribbled in my notebook, didn’t write the date).	• Research suggests that burnout is associated with spending less than 20% of one’s time on the activity that is most meaningful to him or her [83]. • **The Challenge-Hindrance Framework** from psychology posits that not all “stress” is bad: Demanding activities that are perceived as fulfilling (“challenges”) are associated with beneficial outcomes and life satisfaction while demanding activities that are perceived as time-consuming but meaningless (“hindrances”) are associated with negative outcomes and low life satisfaction [84–87]. • **“Attentional residue”** between switching focus from one job demand to another suggests that **multi-tasking** may erode work quality [31, 88, 89]. • In the illuminating article ***Are Academics Irrelevant?***, Randy Stoecker argues that community-engaged researchers cannot and should not expect or be expected to have all the requisite skills to fill all of the necessary roles, such as animator and community organizer [18]. To be relevant and genuinely helpful to a community, Stoecker suggests that researchers should fill only the **roles that reflect their skills**.

Indicating an opportunity to look beyond (post-) positivist paradigms, emergent research suggests that mundane, hidden tasks fuel the implementation process [90]. However, there has been a blind spot to the effect of researchers’ “situatedness,” including intrapersonal resources, values, and ideologies [91, 92], within this hidden dimension of implementation. Moreover, current literature does not include day-to-day, concrete strategies that researchers can use to acknowledge and leverage their situatedness and, therefore, optimally cope with professional and personal challenges within participatory approaches [9]. There is a need to critically investigate the ground-level work within the lived experience of participatory research [11] in order to unpack the black box of implementation [93], including how our own conscious or unconscious situatedness as researchers influences the implementation process.

Therefore, the aim of this research was to explore and analyze what happened in 1 year of a participatory research project dedicated to maternal well-being. For this exploration, researchers employed a novel autoethnographic approach. Autoethnography appears to be a relatively untapped methodology in implementation science [10] and offers a fluid, qualitative approach to capturing the nuanced, inter- and intra-personal micro-processes of how a participatory effort unfolds within an unpredictable real-world, community context [24, 25, 94]. We argue that autoethnography is an appropriate method to investigate current implementation blind spots such as researchers’ situatedness. In combining first-person and third-person perspectives, autoethnography may capture the impact of blind spots and situatedness on all aspects of research from stakeholder interactions to data analysis and interpretation

In exploring nuanced processes, autoethnography combines two central components of science—observation and critical thinking [93, 95]. What autoethnography lacks in conventional control and a priori systemization is made up for by ongoing, interdisciplinary, theoretical dialogue, and critical reflection. Critical reflection [96–98] has not been explicitly explored as a method of implementation inquiry above and beyond the standard integration of reflexivity within qualitative studies. However, in fields such as education and cognitive science, critical reflection is practiced and studied as a process “that allows us a rigorous approach to the scientific work…because it enhances the capability of the mind to go in depth in its life” [98] (p. 7). Intentionally embracing such a subjective method within implementation science poses various limitations, particularly within dominant (post-) positivist paradigms. Therefore, the discussion section includes an assessment of credibility, transferability, dependability, and confirmability as measures of trustworthiness [24, 99, 100].

**Fig. 1:**
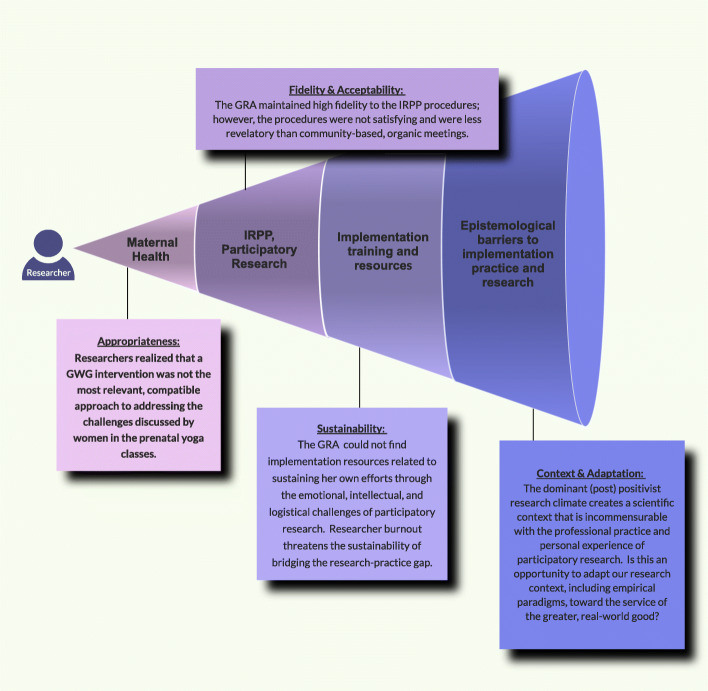
Autoethnography: informing implementation processes and capturing implementation outcomes [101, 102]

This work is significant for three primary reasons. First, it dives beneath the current mechanistic understanding of participatory research by attempting to capture and narrate the complete story and lived experience of implementation, including cognitive-social-emotional phenomena that may elude quantitative methods and current paradigms. Second, the autoethnographic approach may be replicated in other implementation investigations as a rigorous and meaningful method for studying contexts, processes, and outcomes, as depicted in Fig. 1. Third, this work informs actionable strategies for personal stability and empirical depth within the chaos of implementation.

## Methods

An integrated research-practice partnership (IRPP) is a strategic collaboration of stakeholders and researchers to plan, implement, evaluate, and sustain local evidence-based programs [103–106]. In 2009, when the primary investigator (PI, the senior author) was a graduate research assistant learning a specific IRPP approach, she established an IRPP in Southwest Virginia (see Fig. 1) to support the development, delivery, and evaluation of a gestational weight gain (GWG) control program [107]. However, due to high turnover among IRPP members and the departure of the PI who accepted an international postdoctoral position, progress on implementing the GWG control intervention came to a halt. This reflects the common phenomenon of “parachute” research [108, 109] in which researchers disappear from a community setting when empirical work is complete or academic demands arise.

From 2014 to 2018, after completing the postdoctoral fellowship, the PI re-established her community connections and conducted foundational collaborative work on preconception weight management. In 2018, the PI recommitted to prenatal health interventions, pursuing her passion for disseminating evidence-based health information and self-compassion practices to individuals within the gestational period. The graduate research assistant (GRA) whom she hired to manage and evaluate a new IRPP is the first author of this manuscript and conducted autoethnography of her experience implementing the IRPP from July 2018 to July 2019.

In conducting autoethnography, the GRA served as a participant observer [94, 110] simultaneously leading and studying the IRPP. The GRA recorded real-time observations of meetings, interactions, and events as “jottings” which she subsequently expanded into field notes [111]. The GRA wrote field notes for a minimum of 10 min after IRPP-related meetings and events. The GRA also collected cultural artifacts such as meeting agenda, meeting minutes, flyers, and emails [24, 93, 111] and systematically tracked attendance at meetings, start/end times of meetings, adherence to meeting agenda, adherence to communication protocols, and a detailed timeline to measure temporality of implementation efforts and strategies [23].

To capture her own cognitive-social-emotional experience within the work, the GRA wrote general field notes for at least 1 h per week as a process of critical reflection and self-inquiry in which she “play[ed] at the same time the role of subject who reflects and object who is reflected” [98] (p. 1). True to theoretically informed ethnography [110], she iteratively integrated evidence-based concepts and classical theories from other fields [112] so that the fieldnotes were simultaneously data collection and data analysis. This ongoing interdisciplinary [113], “ethnographic-theoretical dialog” [110] is rooted in qualitative research paradigms that emphasize the value-laden, fluid nature of reality (ontology), and knowledge (epistemology) [19, 20, 25].

To capture her own cognitive-social-emotional experience within the work, the GRA wrote general field notes for at least 1 h per week as a process of critical reflection and self-inquiry in which she “play[ed] at the same time the role of subject who reflects and object who is reflected” [99] (p. 1). True to theoretically informed ethnography [110], she iteratively integrated evidence-based concepts and classical theories from other fields [112] so that the fieldnotes were simultaneously data collection and data analysis. This ongoing interdisciplinary [113], “ethnographic-theoretical dialog” [110] is rooted in qualitative research paradigms that emphasize the value-laden, fluid nature of reality (ontology), and knowledge (epistemology) [19, 20, 25].

To organize and further analyze the ethnographic-theoretical dialog, investigators (i.e., the PI and GRA) used a chronological approach called restorying [94] to identify epiphanies or key events within the data and synthesize them into a narrative with the five elements of literature—characters, setting, conflict, plot/action, and resolution. From these elements, investigators generated, interpreted, and presented themes and practical implications of the narrative.

The investigators obtained consent from all IRPP members and protocol approval from the university Institutional Review Board. The GRA’s approach to this work reflects her Master’s of Public Health, academic training in journalism and human development, 15 years of leadership experience as a collegiate swim coach, and ongoing study of yoga. An experienced yoga teacher and practitioner, the PI’s approach to this work incorporates her empirical and practical expertise in pragmatic methods, group dynamics, community-based physical activity, and implementation science. Both investigators were full, active members of the IRPP and neither investigator is a parent; therefore, the investigators simultaneously managed “insider” and “outsider” roles [46, 109, 114] within this participatory project aimed at maternal well-being.

## Results

The presentation of autoethnographic results is a result in and of itself and evidence of the difficulty operationalizing novel methods within current scientific norms such as the common format of peer-reviewed journals. In this project, separating data and interpretation may dilute the reflective, iterative nature of ethnographic-theoretical dialogue. Therefore, investigators suggest that readers iteratively refer to Table 1 throughout the “Results” and “Discussion” sections to experience the visceral-intellectual rhythm of autoethnography and critical reflection. In Table 1, investigators present results of the ethnographic-theoretical dialogue, and in the subsections below, investigators report the results of restorying, including plot, setting, characters, conflicts, themes, and resolution.

### Restorying: plot and setting

**Fig. 2:**
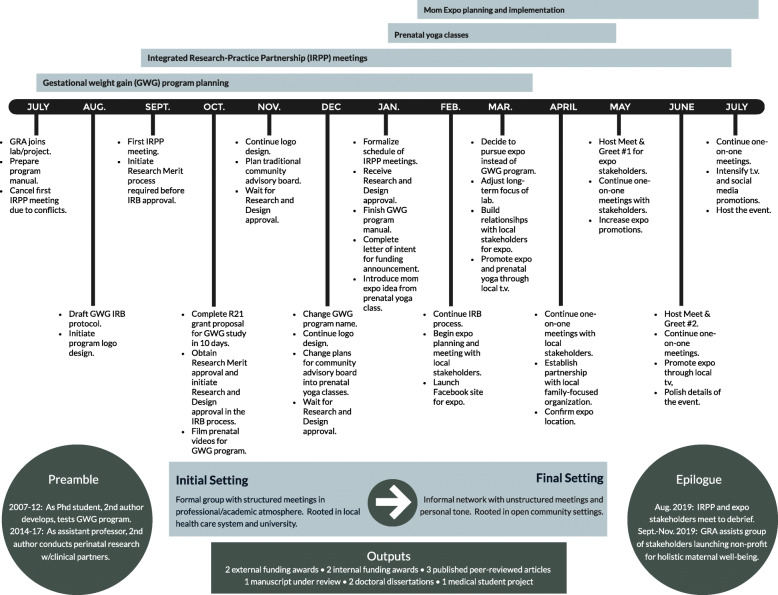
Restorying the plot and setting of the IRPP, July 2018–July 2019

**Fig. 3:**
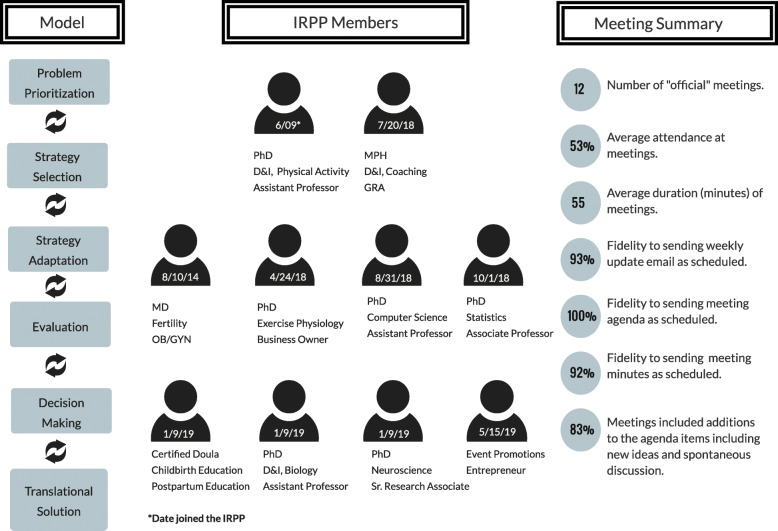
IRPP model, member description, and process

The IRPP’s narrative is set in Southwest Virginia where the connection between a public land-grant university and local health care system developed over more than a decade. As depicted in Fig. 2, the IRPP’s “plot” within this setting dramatically shifted from July 2018 to July 2019. In July 2018, the IRPP convened to plan a randomized controlled study (RCT) of a GWG control program. Investigators aimed for the study to be a systematic step in “scaling out” [115] the program by delivering it through videoconference. To adapt the program, the GRA intended to develop a community advisory board as an additional arm of the IRPP. As the investigators worked with local women to film prenatal physical activity videos, the plans for a community advisory board morphed into free prenatal yoga classes. Attendees of the prenatal yoga classes suggested the idea of a 1-day, family-friendly event for local women. This idea developed into the planning, implementation, and pragmatic evaluation of a maternal health fair to empower, educate, and connect local mothers in July 2019. The event featured 23 local educators including a lawyer, physical therapist, music therapist, and neuroscientist, who presented interactive workshops including “Self-Advocacy at Home, Work, and Your Doctor’s Office,” “Pelvic Floor Exercises for Pregnancy and Postpartum,” “Music for Labor Through the Early Years,” and “Mommy Mind” (manuscript under review). See Fig. 3 for the structure and composition of the IRPP in the year leading to the maternal health fair.

Within data collection and analysis, it was apparent that understanding the IRPP’s “plot” required looking beyond the 12-month autoethnography: “The IRPP isn’t in a petri dish. [The PI] has been working on [the GWG program] since she was a graduate student and there’s already discussion about how this is just the first Mom Expo [the maternal health fair], just one step toward better connections for pregnant and postpartum women. When I get caught up in the 12-month time frame of the protocol, I forget that the IRPP actually exists on a continuum of time and life with all sorts of inputs and outputs. It’s not like a carefully prepared sterile slide under a microscope” (field notes, June 6, 2019). In other words, what led to this IRPP and what stems from it extends before and after the empirical, researcher-established boundaries of a project (see preamble and epilogue in Fig. 2).

Within this unbounded “setting,” seemingly mundane interpersonal interactions rippled into long-term effects. For example, the GRA established a connection with a local community member who attended a single prenatal yoga class in March 2018. That community member provided an introduction to a leader of an existent local non-profit organization who agreed to host the first event of the new non-profit organization that developed from the maternal health fair that originated in the yoga classes. In other words, a single interpersonal relationship that developed within a prenatal yoga class that was not part of the initial IRPP plan contributed to the significant, unpredicted long-term community benefit of a non-profit organization for holistic maternal well-being. Likewise, other interpersonal connections the GRA developed within the community led to promotional spots on local television stations and reduced financial costs for hosting the maternal health fair. “This whole thing is like the butterfly effect: I don’t know how or when I meet someone will lead to something else later down the road” (field notes, July 28, 2019). The butterfly effect, first demonstrated by Edward Lorenz in mathematical models of weather predictions, describes how seemingly inconsequential events eventually contribute to significant outcomes in a later state [116], as in the flapping of a butterfly’s wings in Brazil causing a tornado in Texas [117]. The butterfly effect provides a metaphor for the dynamic, unpredicted effects of spending time with community members within informal settings that belie conventional scientific protocol.

Based on the GRA’s initial D&I science training in a graduate course, she expected “what happens in an IRPP” to be a slow but steady, systematic but iterative flow. “The language about implementation frameworks, theories, and processes from D&I class implies a slow but continuous spread: *diffusion* [italics included] of innovations, 17 years to move through the traditional pipeline from basic science to uptake in the real world, iterative and ongoing balance of fidelity and adaptation. It sounds very Darwinian—gradual evolution, survival of the fittest. Even the language of a diffusion ‘champion’ conjures up an image of the ‘fittest’” (field notes, December 9, 2019). However, in terms of metaphors from natural science, the full IRPP story resembles punctuated equilibrium (PE) [118]. PE is a model of evolution that describes change patterns in nature as occurring through periods of relative stability suddenly punctuated by rapid adaptation [118]. Social science researchers have recently used this evolutionary model to describe the delayed benefits of coaching when a certain event triggers an epiphany that catalyzes an individual to suddenly enact what he or she previously learned in coaching [119]. Likewise, evaluation scientists have highlighted “evolutionary epistemology” and posited that the natural selection of what knowledge survives and spreads in the real-world is not a “foreordained” process but takes place in “fits and starts” [120] (p. 4). These fits and start are evident in the full story of the IRPP which spans chunks of “latent” time punctuated by blocks of intense activity in 2008–2009, 2011, 2014, and 2018–2019. Even within 2018–2019, there was a distinct shift in pace (i.e., a punctuation) when the prenatal yoga classes catalyzed action toward the maternal health fair. The catalysts for the action were not the mechanistic operations of the IRPP, but the characters and conflicts within the story.

### Restorying: characters, conflicts, and themes

On the surface, the PE pattern of IRPP-related work was contingent upon investigators’ situatedness as graduate students: The highest intensity blocks occurred during 2008–2009 when the PI initiated the GWG program and community partnerships as a graduate student, and during 2018–2019 when the GRA managed the IRPP and founded the health fair. Graduate education resources such as predoctoral fellowships, protected time, and supervision from experienced advisors enabled intense empirical and practical activity: The investigators’ “situatedness” [29] as graduate students set the stage for generating ideas, completing time-intensive research, and establishing extensive personal networks through a participatory approach.

Beneath the concrete academic resources, the investigators’ lived experience demonstrated their situatedness as human beings [109] with ever-present emotions, deep values, limited time, and finite energy making them vulnerable to strain [121] from work demands. For example, the GRA experienced emotional exhaustion and ego depletion [72, 73] stemming from a complicated web of her own associative thinking [71], maladaptive perfectionism [69, 122], and perseverative cognition [50, 51] that was exacerbated by a publish or perish research culture [74, 123] rooted in extrinsic motivation [75, 76] and performance achievement orientation [124]: “All year I’ve felt like I’m on a wild-goose chase or playing some Alice-in-Wonderland version of dominoes, balancing what a dissertation committee values as science with what prenatal yoga women value as life, all while keeping up a can-do attitude and A+ quality amidst the constant topple of…’*let’s change the name for [the GWG program]’…’actually, let’s not do the [GWG program]’*…*’you have to have a research question’*” (field notes, March 25, 2019). The autoethnographic process itself helped the GRA recognize how her own cognitive patterns contributed to her exhaustion, and subsequently, she was able to reshape maladaptive rumination and over-analysis into insightful reflection [125, 126].

Critical reflection within this research design illuminated dissonance between the investigators’ professional, conventionally scientific roles and their person-centered participatory roles. The difficult decision to pivot away from an RCT epitomized this conflict: In implementing a pragmatic maternal health fair instead of an intervention RCT, the investigators intentionally chose to prioritize participatory input over traditional a priori empirical processes: “I can’t truly empower people in the spirit of participatory research if the only way to achieve ‘rigor’ is to march steadily forward on a pre-fixed, ‘systematic’ path based on *a priori* research questions, not on the destination women really seek” (field notes, March 13, 2019). In addition, this shift manifested investigators’ epiphany that GWG was not local mothers’ central need: “…we get caught up in creating band aids, not treating wounds. Bandaids are kind of like packageable, tidy interventions for narrow health outcomes, like GWG, that are important but not the real source of suffering, not the central thing that heals the core issue or fuels all-around well-being…To achieve something *sustainable* that people want to *maintain* with *fidelity*, we have to get at the underlying root issue and what really matters to people, even if it’s hard to measure.” (field notes, February 24, 2019).

With this epiphany, the GRA’s work expanded from leading structured IRPP meetings to spending one-on-one time with stakeholders out in the community in non-academic, non-clinical settings such as coffee shops and libraries. From July to March, the IRPP had inched toward launching the RCT as investigators grappled with organizational red tape and convoluted procedures within the clinical system: “Busyness gets in the way of business. There’s tons of horizontal movement—as in digital horizontal movement as emailsemailsemails gobble up the day—but no vertical progress toward the mountain peak” (field notes, October 14, 2019). In contrast, progress toward the health fair was rapid, including partnerships with an existing family-oriented organization and local library, as well as commitments from nearly 40 individuals to volunteer their service, expertise, and energy at the health fair. “I get so much more input from one-on-one get-togethers out in the community then when I run IRPP meetings with agenda and minutes and action items. The IRPP meetings are systematic and trackable; but for all the organization (i.e. replicable processes worth publishing in a journal), they don’t seem nearly as authentic as the one-on-one meetings with women out in their community…where I’m making traction, where I feel like I’m gaining insight into community dynamics, where relationship-building doesn’t feel like a mercenary research task” (field notes, April 12, 2019).

**Fig. 4:**
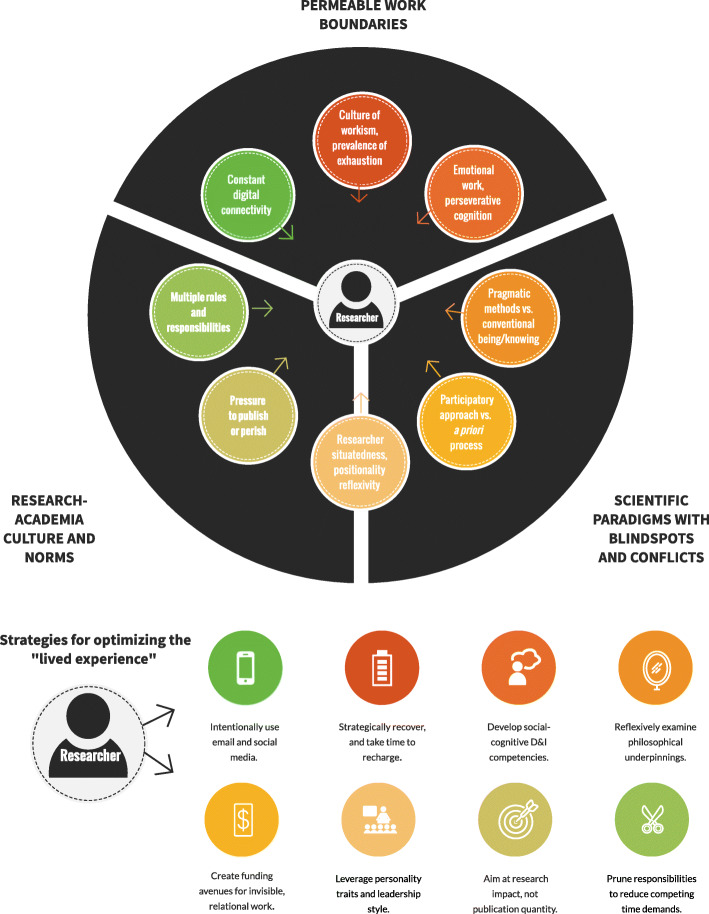
Themes of the researcher lived experience

In living out these epiphanies and conflicts, investigators identified three themes: (1) permeable work boundaries [127], (2) individual and collective blind spots toward the ontological and epistemological underpinnings of implementation paradigms, and (3) maladaptive behaviors seemingly reinforced by the research culture. See Fig. 4 for a diagram of the themes and Table 1 for subthemes, ethnographic evidence, theoretical/literature dialogue, and untapped opportunities for progress.

One lens for interpreting these intertwined themes is structuration theory [128] which “refers to the idea that rules, policies, and structures are only made ‘valid’ when individuals follow them and make decisions based upon them” (p. 59) [25]. In other words, the GRA’s own behaviors—her individual “micro-practices”—reinforced the publish or perish culture and (post-) positivist paradigms that may hamper participatory research and well-being. For example, in the first 6 months of the project, the GRA’s drive for achievement reinforced her situatedness in a culture of workism [127]: “I was tossing deep thinking out the window so that I could keep driving forward as fast as possible…my ambition to be more ‘productive’ devoured my responsibility for self-care” (field notes, December 27, 2018). But as the IRPP work turned to relationship building in the second 6 months, the GRA found some traction by distinguishing between presence and productivity: “I cannot control all of the variables that go into scientific productivity, but I can control where and how I choose to focus my energy…the depth and deliberateness of my presence in each and every conversation even if I cannot control an editor’s or committee member’s reaction to a paper I write” (field notes, March 21, 2018). This distinction did not lead to a mindful panacea of constant ease. Instead, it has led to unease knowing that challenging the prevalent implementation *structure* may lead to conflict and rejection within the field.

The field has made significant advancements in developing toolkits to clarify empirical outcomes of implementation [101], establish skill-based mentoring [48], and promote qualitative methods that capture nuanced micro-processes [24]. Furthermore, at the 2018 and 2019 Annual Conferences of the Science of Dissemination and Implementation in Health, there were calls to acquire “soft” skills. However, we have yet to appropriately operationalize these calls and advancements by determining what roles an implementation scientist should or could realistically play within participatory research [18] based on their unique skills, training, personality, values, and time—in other words, based on their situatedness and finite human capacity [73]. The lack of awareness or reticence to recognize personal situatedness and limits of human capacity [73] may be a result of traditional scientific paradigms that dominate health research: The structure of grant writing [58], system of tenure (cultural artifact, university tenure guidelines), emphasis on outcomes [102], and alignment with conventional benchmarks of rigor such as objectivity and internal/external validity [7, 129, 130] indicate dominance of (post-) positivism [19, 20, 25]. Likewise, the general style of reporting results in peer-reviewed journals reflects a paradigm of value-free objectivity. Researchers generally write methods sections in a passive voice (e.g., “data was collected…”); avoid the subjective pronouns *I*, *we*, and *you*; and describe procedures that are measurable and replicable. Minimizing attention to the inherent role of their own five senses and cognition, researchers often un-situate themselves as an indication of unbiased validity and reliability. This paradigm of projecting machine-like expertise seems incommensurable with both reflective practice [96, 97] and participatory research which hinges on researchers’ moral drive to make a difference in the real-world and their ability to build trust with stakeholders [131]. Building trust is a human—not scientific—venture, and this ethnographic-theoretical dialogue suggests that researchers’ humanness—for example, being “too tired to inspire” [64]—is a critical component of the black box of implementation. Furthermore, researchers’ deep philosophical views and scientific training may limit their perspective on novel ways to open that black box. For example, authors of a recent implementation autoethnography observed an “underlying tension between the production and use of generalizable knowledge” caused by conflicting scientific paradigms [10] (p. 6). This paper avoids that trap by elucidating strategies to uncover paradigm conflicts that reduce the usability of findings (see the “Conclusion” section).

Fields such as education and mindfulness have embraced debates on the ontological and epistemological foundations of research and practice [97, 98, 132–134], and the emerging idea of value-based medicine [135, 136] emphasizes philosophical underpinnings and patient empowerment [20]. However, implementation dialogue focuses on the methodological level of what constitutes credible evidence, not philosophical viewpoints of reality and knowledge [55]. The lack of clarity on these ontological and epistemological levels may contribute to the chaos [17] and lexicological quagmire [8, 112, 113, 137] often described within implementation science. A firm footing in the field’s beliefs about science may provide a foundation to stabilize at least some of the chaos: “Time and time again, I’ve seen that the swimmers who consistently win aren’t always the ones with the most efficient stroke or highest V02 max—it’s often the ones who had the strongest *why*, the clearest perspective of what a race meant, a purpose beyond the numbers on the record board…” (field note, February 2, 2019). Paradoxically, the coaches and athletes who win the most are often the ones who anchor their focus on a purpose beyond winning, beyond tangible outcomes measured by a stopwatch or counted in a win-loss record [138, 139]. In contrast, the GRA observed an overemphasis on outcomes over process and methods over philosophy within the IRPP, implementation literature, and academic research environment. It was through the *process* of autoethnography that the GRA unearthed her own ontological and epistemological assumptions; she experienced the effectiveness of reflective practice to clarify her own philosophy as a stabilizing point within the messiness of implementation.

### Restorying: resolution

The resolution of the 12-month study indicates empirical, practical, personal, and philosophical evolution. On the empirical level, the GWG control intervention evaluated through RCT evolved into a health fair evaluated through the pragmatic application of the RE-AIM framework [140] (manuscript under review). On the practical level, the GRA’s work evolved from leading an IRPP in a systematic way to meeting community members for coffee and “walk and talks.”

On the personal level, continuous reflection through this methodological approach prompted the investigators to intentionally shift their behaviors and micro-practices to optimize their own situatedness including well-being and creativity: The PI practiced delegation and “allowed” the IRPP, health fair, and non-profit to form organically, adjusted research focus in order to avoid emotional triggers, committed to eating lunch before 3 pm, crafted a value-laden mission statement for the lab, and carved out time away from email. The GRA re-vamped her morning breathing meditation practice, thwarted work-related rumination by listening to unrelated podcasts during her commute, and stepped away from the computer to hug her husband whenever he arrived home. In line with the Conservation of Resources theory [120, 141] from industrial and organizational psychology, these shifts helped optimize situatedness by conserving and refueling researchers’ intrapersonal energy and interpersonal resources to meet the demands of participatory implementation research.

Finally, at the philosophical level, the GRA evolved from focusing on novel *methodologies* to grappling with deeper ontologies and epistemologies that influence or limit implementation science and participatory research. Paradoxically, the use of a novel methodology (i.e., autoethnography) is what revealed that novel methodologies are not enough: There is a need to examine and align underlying ontological and epistemological foundations of implementation science, including the roles of subjectivity and values within participatory research. PE paleontologists Eldredge and Gould embraced subjectivity within the scientific process when they wrote, “We do not encounter facts as *data* (literally “given”) discovered objectively. All observation is colored by theory and expectation” [118] (p. 85). With a similar nod toward embracing bias, the investigators report the trustworthiness of this study in Table 2. In this paper, we have transparently presented our own vulnerabilities and biases as examples of how researcher situatedness may impact participatory approaches to implementation. In addition, we have leveraged interdisciplinarity to interpret these vulnerabilities as untapped opportunities to advance the field of implementation science.

**Table 2:** Reporting trustworthiness of qualitative data based on Guba and Lincoln’s criteria [142]

Dimension	Evaluation
**Dependability:** consistency and accuracy of findings	Through iterative, ongoing immersion in peer-reviewed literature from a variety of fields, we anchored the ethnographic observations in scientific findings. Essentially, we demonstrated that researchers and participatory implementation processes are consistent with concepts from the social sciences and natural sciences.
**Credibility:** truth of the findings	Our reflexivity statements in the methods section and transparency with which we approached our own struggles and situatedness indicate that the findings are true to our lived experience within this IRPP.
**Confirmability:** neutrality and whether findings are supported by evidence	The findings suggest that we were not neutral and emotionless during the participatory process; however, we transparently (and vulnerably) reported our own holistic involvement in the process, triangulating data from cultural artifacts, the GRA field notes, collaborative reconstruction of events, and ongoing theoretically informed brainstorming.
**Transferability:** application in other contexts	We report our unique lived experience within a unique IRPP and do not expect our exact thoughts, emotions, and relationships to be replicated in other contexts. However, insight to the importance of situatedness is transferable to other researchers. Regardless of the scientific paradigms to which we ascribe or objectivity to which we strive, other researchers can also leverage the transformative potential of reflection to elevate personal meaning, practical relevance, and empirical depth.

## Discussion

Based on this study, we propose that implementation researcher situatedness influences all aspects of participatory research from planning to dissemination. Situatedness is multifaceted with personal (i.e., the researcher), interpersonal (i.e., the researcher + IRPP), and cultural (i.e., the researcher + implementation science) dimensions that include conscious and unconscious, adaptive and maladaptive drivers. It stands to reason that these drivers influence many manifestations of implementation science, not just participatory research. Therefore, the implications of this work are applicable to all implementation scientists.

**Fig. 5:**
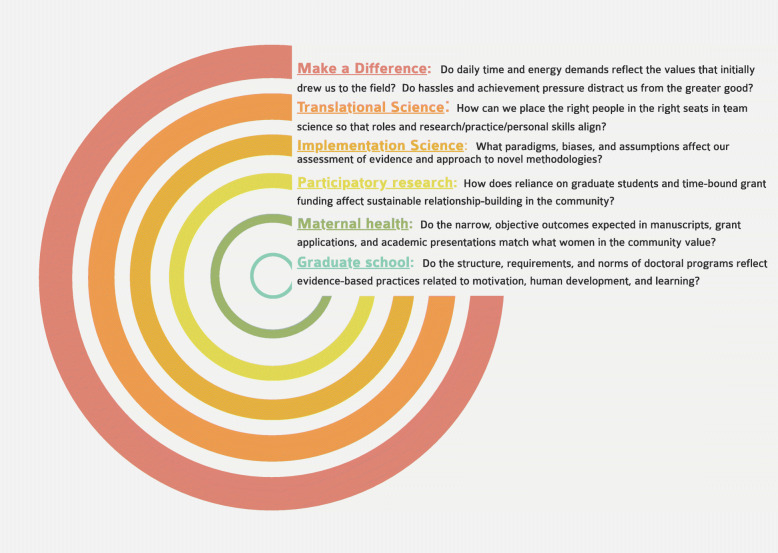
Frames of reflection within the autoethnography

This work indicates that autoethnography is a thought-provoking, action-inspiring method to leverage researcher situatedness through critical reflection, capturing empirical aspects of implementation while enhancing the practice of implementation including researchers’ own influence on the process. As shown in Figs. 1 and 5, the reflective nature of autoethnography can deepen researchers’ understanding of implementation contexts, processes, and outcomes [101, 102] and generate questions within radiating frames of reference. In addition, autoethnography brings interdisciplinary [113] work to life by facilitating the identification and application of theories from other fields, particularly social science [143] where researchers have grappled with real-world messiness for years [144]. Future work in implementation science is needed to distinguish between empirical autoethnography and “mere anecdotes” [97] (p. 198) and to develop strategies for disseminating the breadth and depth of data captured in this time- and energy-intensive method.

Findings in this explicitly reflective work suggest that there are gaps in acknowledging our own humanness and in investigating “invisible” drivers such as deep ontological and epistemological foundations of implementation science. Without deeper recognition of the ontological and epistemological components of the current implementation paradigm, new methodologies will fall flat; we will not be able to clear the research-real world pipeline that we inadvertently clog through our own attachment to traditional views of what matters and what is. Furthermore, we may exacerbate gaps and blind spots by unintentionally behaving in maladaptive ways that reinforce the culture of workism, demonstrable productivity, and chaos. Within this chaos, we suggest several strategies to optimize situatedness.

First, we suggest incorporating ontological and epistemological inquiry into academic training, team science, and efforts to advance the implementation field. Diving beneath the current focus on matching methods to a research question, researchers should reflect on personal philosophies that underscore or contradict paradigms of what evidence counts [55]. See Table 3 for potential benefits of acknowledging philosophical underpinnings.

**Table 3:** Potential benefits of recognizing and clarifying ontological and epistemological paradigms

• Improve **communication across the translational spectrum** from basic science to clinical science to implementation, dissemination, and policy.	
• Leverage **the power of values and intrinsic motivation in fostering long-term change**, as opposed to relying only on impersonal, measurable mechanisms that do not reflect basic human psychosocial needs such as love, connection, and autonomy [76, 145].	
• Engage in **rich analysis of novel approaches** that may otherwise be quickly rejected because of incongruence with the analyzer or field’s deeply held, implicit views as what counts as evidence or knowledge.	
• **Advance interdisciplinary and transdisciplinary approaches** [113] that may be initially discarded because of underlying, mismatched philosophical assumptions between various fields.	

Second, we suggest that researchers adopt evidence-based restorative practices that align with one’s own values, similar to how elite athletes prioritize recovery in their training regimens [146, 147]. Potential practices include microbreaks [148, 149], meaningful off-job experiences [150], slow deep breathing [151, 152], and breaks from digital communication [153].

Third, to promote presence, we suggest that researchers focus their senses, intellect, and emotions on the people present in a specific time and place: Acknowledge the potential of “n-of-1” participatory research in which individual, authentic relationships can spiral into unpredicted community benefit in a butterfly effect pattern. Embrace personal presence as a valuable experience in and of itself in addition to being a vehicle for relevant empirical productivity.

## Conclusion

Each of these strategies incorporates reflective practice and values as stabilizing mechanisms within the chaos of implementation: Clarity through the chaos of implementation starts with clarity within the researcher—as a situated human being viscerally and cerebrally experiencing life. We may not be able to control dynamic, real-world variables and unpredictable external contexts, but we can choose to maximize our humanness and presence. In leveraging our own situatedness through critical reflection, we can apply implementation-related constructs to ourselves: *scale* inward, *facilitate* one’s own capacity, protect personal *sustainability*, seek *fidelity* to values, and embrace the present *context* of moment-to-moment time and space. Ultimately, through this critical reflection, implementation scientists may epitomize what Margaret Mead meant by *thoughtful* citizens changing the world.

## Data Availability

Publicly sharing all autoethnographic data, including all field notes and cultural artifacts, may compromise personal privacy. Table 1 and the extensive integration of field notes throughout the manuscript provide the minimal data needed to interpret, replicate, and build upon findings reported in this article.

## Abbreviations

D&I: Dissemination and implementation
GRA: Graduate research assistant
GWG: Gestational weight gain
IRPP: Integrated research practice partnership
PE: Punctuated equilibrium
PI: Principal investigator
RCT: Randomized controlled trial
RE-AIM: Reach, effectiveness, adoption, implementation, maintenance
V02 max: Volume of oxygen consumption during maximal exercise
